# FMT from IBS-D Rats Impairs Intestinal Barrier Function and is Associated with Increased Intestinal Benzoic Acid and *Ruminococcus* Abundance

**DOI:** 10.4014/jmb.2602.02004

**Published:** 2026-06-04

**Authors:** Xiangyu Xie, Weixin Yan, Xinting Zhang, Chunli Gan, Xiaochun Lei, Yusheng Huang, Wei Xiong, Hongmei Tang, He Zhu

**Affiliations:** 1 The First Clinical School of Guangzhou University of Chinese Medicine, Guangzhou 510405, P. R. China; 2 The First Affiliated Hospital of Guangzhou University of Chinese Medicine, Guangzhou 510405, P. R. China; 3 Zhongshan Hospital of Traditional Chinese Medicine, Zhongshan 528400, P. R. China

**Keywords:** IBS-D, Fecal microbiota transplantation, Gut microbiota, Gut microbiota metabolism, Benzoic acid, *Ruminococcus*

## Abstract

While the etiology of diarrhea-predominant irritable bowel syndrome (IBS-D) is multifactorial, current studies have converged to identify gut microbiota dysbiosis as a principal contributor. This study aimed to elucidate the role of the gut microbiota in IBS-D progression and to uncover the underlying mechanisms. In this study, a rat model of IBS-D was successfully established, characterized by prominent visceral hypersensitivity and diarrhea. To assess the impact of the gut microbiota, recipient rats pretreated with broad-spectrum antibiotics underwent fecal microbiota transplantation (FMT) from IBS-D model rats. The model-microbiota recipient rats (the MR group) developed IBS-D-like symptoms, such as abdominal pain, diarrhea, and depression-like behaviors. Both the IBS-D and MR groups exhibited elevated serum diamine oxidase (DAO) concentrations, reduced intestinal tight junction protein levels, increased serum TNF-α concentrations, upregulated TNF-α mRNA expression, and downregulated IL-10 mRNA expression in the intestine. These findings indicated that the transplanted microbiota disrupted intestinal barrier integrity and triggered low-grade inflammation. Moreover, an elevated abundance of *Ruminococcaceae* in the gut microbiota was a common feature of both the IBS-D and MR groups. Metabolomic analysis revealed an enrichment in the phenylalanine, tyrosine, and tryptophan biosynthesis pathway in both IBS-D and recipient groups, with benzoic acid being particularly prominent. Pearson correlation analysis demonstrated a strong positive correlation between the *Ruminococcus* abundance and benzoic acid levels. Together, these findings indicated that FMT robustly recapitulated core IBS-D pathophysiology in recipient rats, and the identified bacteria and metabolites provided novel insights into the pathogenesis of IBS-D.

## Introduction

Irritable bowel syndrome (IBS) is a functional gastrointestinal disorder characterized by abdominal pain and bowel habit abnormalities, and often accompanied by emotional disturbances [1]. However, its clinical diagnosis remains challenging due to the absence of reliable and quantifiable biomarkers [2, 3]. The diarrhea-predominant IBS (IBS-D) is the most prevalent, accounting for approximately 30% of reported cases [4, 5]. Its pathogenesis is complex and multifactorial, involving key elements such as gut microbiota dysbiosis, visceral hypersensitivity, increased intestinal permeability, and low-grade inflammation [6].

The gut microbiota comprises approximately 10^14^ microorganisms and significantly influences the host’s physiological functions, earning it the nickname “the second brain” [7]. Gut microbial dysbiosis has been implicated in diverse systemic conditions, including diabetes, alcoholic hepatitis, and intestinal health [8-10]. Through the microbiota-gut-brain axis, these microbial communities play an instrumental role in the pathogenesis of intestinal, metabolic, and neurological disorders [11], prompting the hypothesis that compositional changes in the microbiota may be central to symptom development.

With advancements in gene sequencing technology, researchers have gained deeper insights into the gut microbiota. Clinical studies demonstrate that IBS patients showed significant alterations in their gut microbiota composition, characterized by increased abundances of *Enterobacteriaceae*, *Lactobacillaceae*, and *Ruminococcus*, which were potentially associated with symptom severity and disease progression [12-14]. These clinical observations regarding both specific microbial changes and variable diversity patterns aligned with and support findings from our experimental animal models of IBS. However, there was an inconsistency across studies regarding microbial diversity in IBS. While some reported a reduction in α-diversity, others found no significant difference relative to healthy controls [15, 16]. This discrepancy may be due to factors such as regional differences and dietary habits, leading to the absence of a consensus on the specific microbial shifts in IBS-D patients [17]. Recent studies have investigated the link between IBS-D phenotypes and the gut microbiota. Notably, evidence from fecal microbiota transplantation (FMT) experiments confirmed that gut microbiota can actively modulate host intestinal behavior and immune responses [18]. Furthermore, researchers have reported that gut microbiota directly contact mast cells [19]. Such contact could potentially explain the underlying cause of immune activation and visceral hypersensitivity reactions in IBS-D. Despite this mechanistic insight, the specific microbial biomarkers and their associated metabolic pathways in IBS-D remain incompletely understood.

An IBS-D rat model was established for this study, and FMT was utilized to examine the effects of donor feces on intestinal pain threshold, fecal water content, inflammatory markers, and barrier integrity in recipient rats. We further characterized the gut microbiome and microbiota-associated metabolome profiles in both IBS-D donor and FMT-recipient rats.

## Materials and Methods

### Chemicals and Reagents

*Sennae folium* (batch number: 230701) was purchased from Zhixin Traditional Chinese Medicine Decoction Pieces Co., Ltd. (China). All antibiotics, including neomycin sulfate (N814740), ampicillin (A830931), metronidazole (M813526), and vancomycin hydrochloride (V820413), were purchased from Macklin Biochemical Co., Ltd. (China).

### Animals

Pregnant rats (300-400 g; certificate Nos. 44007200127124 and 44007200128824) were purchased from the Guangdong Experimental Animal Center [license number SCXK (Yue) 2022-0002]. The rats were housed under SPF conditions at the Experimental Center of the First Affiliated Hospital of Guangzhou University of Chinese Medicine. All procedures were approved by the hospital's Ethics Committee (approval No. GZTCMF1-20230053).

### Induction of the IBS-D Rat Model

The experimental method was based on established protocols, with minor adaptations [20, 21]. Neonatal rats were randomly divided into the Control group and the IBS-D group. Rats in the IBS-D group were subjected to maternal separation for 14 days, with a duration of 3 h per day. Subsequently, the IBS-D group was subjected to 3 hours of restraint stress daily. On the 28th day, the IBS-D rats received a daily gavage of *Senna folium* decoction (5 g/kg) for 7 consecutive days. Body weight was recorded weekly starting on the 21st day. On days 35-36, fecal samples were collected, and fecal moisture content (FMC) [22], the abdominal withdrawal reflex [23], and the sucrose preference test (SPT) [20] were assessed as described. Following these final in vivo assessments, blood was collected from the abdominal aorta under deep isoflurane anesthesia, and subsequent euthanasia was confirmed by cervical dislocation.

### Preparation of Fecal Microbiota Solution

As previously described [24, 25], fecal samples from IBS-D rats were homogenized in phosphated buffered saline (PBS) at a 1:10 (w/v) ratio. Following debris removal, the homogenate was centrifuged at 800 × g and 4°C. After three washes with PBS, the pellet was resuspended in PBS containing 10% glycerol for storage at -80°C. Samples were thawed in a 36°C water bath before use.

### Establishment of Pseudo-Germ-Free Rat Model and FMT

To establish a pseudo-germ-free rat model, normal male rats (28–30 days, 80–100 g) received a 5-day oral gavage of a mixed antibiotic cocktail: neomycin (200 mg/kg), ampicillin (200 mg/kg), metronidazole (200 mg/kg), and vancomycin hydrochloride (100 mg/kg) [26]. Following model establishment, the rats were randomly divided into the PBS group and the model-microbiota receptor (MR) group. The MR group underwent daily rectal administration of 1 ml IBS-D fecal microbiota suspension for 10 days, whereas the PBS group received an equal volume of vehicle.

### Enzyme-Linked Immunosorbent Assay (ELISA)

The serum concentrations of diamine oxidase (DAO) and TNF-α were quantified according to the manufacturer’s instructions using ELISA kits (RA20028, RA20035; Bioswamp, China).

### Quantitative Real-Time Polymerase Chain Reaction (qRT-PCR)

Colon RNA was extracted using TRIzol and reverse transcribed into cDNA. qPCR was performed with SYBR Green reagent (AG11702, Accurate Biology, China) on an ABI QuantStudio5 system (Thermo Fisher Scientific, USA). Primer information is shown in Table 1, and GAPDH expression served as the internal control for relative mRNA expression.

### Western Blotting

After extraction and quantification of colon tissue proteins, 20 μg of protein per lane was separated by SDS-PAGE, transferred onto PVDF membranes, and probed overnight at 4°C with the specific antibodies, including ZO-1 (1:1500, AF5145, Affinity), Claudin-1 (1:1000, 13050-1-AP, Proteintech), Occludin (1:5000, 27260-1-AP, Proteintech), and then incubated with secondary antibodies for 1 hour at room temperature. Bands were detected by the ChemiDoc MP system (Bio-Rad, USA) and subsequently quantified with ImageJ.

### 16S rRNA Sequencing Analysis

The extraction and sequencing services were provided by Novogene Technology Co., Ltd. (China). Briefly, intestinal content DNA was isolated (TIANamp Soil DNA Kit, TIANGEN, China) and the V3–V4 regions of the bacterial 16S rRNA gene were amplified by PCR using the primers (515F: 5’- CCTAYGGGRBGCASCAG-3’, 806R: 5’- GGACTACNNGGGTATCTAAT-3’). The NovaSeq PE250 platform (Illumina, USA) was utilized for 16S rRNA sequencing. Reads from each sample were merged using FLASH (version 1.2.11), followed by stringent filtration using fastp software (version 0.23.1). The DADA2 plugin implemented in QIIME2 (version 202202) was employed to generate the final amplicon sequence variants (ASVs). Taxonomic assignment was conducted using QIIME2 against the Silva 138.1 database. The 16S rRNA sequencing data were uploaded to the NCBI, and the accession number was PRJNA1353073.

### Untargeted Metabolomic Analysis

The extraction and sequencing services were provided by Novogene Technology Co., Ltd. (China). Intestinal content samples were ground, homogenized in 80% methanol, and centrifuged (15,000 × g, 20 min, 4°C). The resulting supernatant was diluted to 53% methanol and re-centrifuged. The final supernatant was collected for LC-MS/MS analysis. Data were analyzed using the Novo Cloud platform (https://magic-plus.novogene.com/).

### Statistical analysis

Data were analyzed using GraphPad Prism 8.0 and are presented as mean ± standard deviation (mean ± SD). Pairwise comparisons were performed using Student’s t-test. Statistical significance was set at *p* < 0.05.

## Results

### IBS-D Microbiota Recipient Rats Developed IBS-D Symptoms

**Fig. 1:**
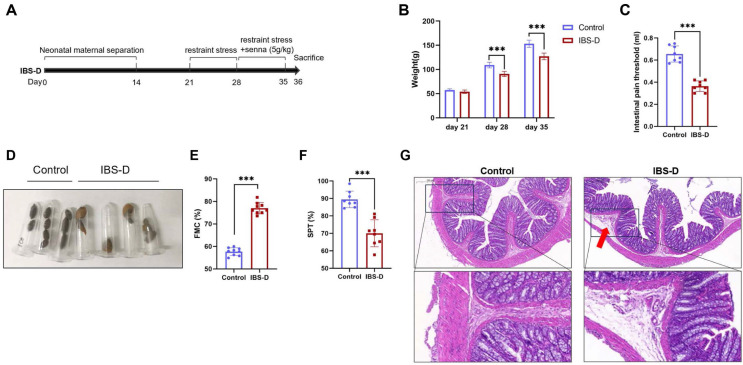
Establishment of IBS-D rat model (n = 8). (**A**) Schematic diagram of the IBS-D rat model. (**B**) Weight changes in IBS-D rats. (**C**) Intestinal pain threshold. (**D**) Diarrhea situation. (E-F) FMC and SPT value in rats. (**G**) Representative images of HE staining (100×). Values are expressed as the mean ±SD; ***p < 0.001.

The IBS-D model was established (Fig. 1A) and body weight was recorded weekly starting from the 21st day. As shown in Fig. 1B, compared with the Control group, IBS-D model rats exhibited a significantly lower body weight. Since abdominal pain, diarrhea, and emotional abnormalities are typical symptoms of IBS-D, we assessed these domains using relevant behavioral and physiological metrics. Successful model validation was confirmed by a reduced intestinal pain threshold, elevated FMC, and a decreased SPT score in the IBS-D group (Fig. 1C-1F).

**Fig. 2:**
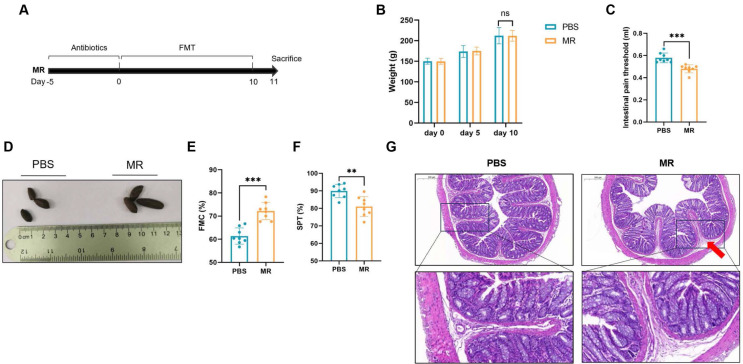
Fecal microbiota transplantation replicated IBS-D symptoms (n = 8). (**A**) Schematic diagram of FMT. (B) Weight changes of fecal microbiota transplantation recipients in rats. (**C**) Intestinal pain threshold. (**D**) Diarrhea situation. (**E-F**) FMC and SPT value in rats. (**G**) Representative images of HE staining (100×). Values are expressed as the mean ±SD; **p < 0.01, ***p < 0.001.

After creating pseudo-germ-free rats using a mixed antibiotic regimen, we performed FMT from IBS-D donor rats to naive recipient rats (Fig. 2A). During routine weight monitoring, no significant difference could be found between the MR and PBS groups (Fig. 2B), indicating that FMT did not impair general growth. In contrast, the MR group exhibited significant alterations in key IBS-D-relevant metrics: a reduced intestinal pain threshold, increased FMC, and a decreased SPT score (Fig. 2C-2F). Histological examination of colon tissue revealed normal histology in the Control and PBS groups. In contrast, the IBS-D and MR groups exhibited occasional submucosal edema (Fig. 1G and Fig. 2G). These results demonstrate that FMT from IBS-D donors successfully induced IBS-D-like symptoms in normal recipients.

### Fecal Microbiota from IBS-D Rats Induced Colonic Permeability Alterations and Low-Grade Inflammation in Recipient Rats

**Fig. 3:**
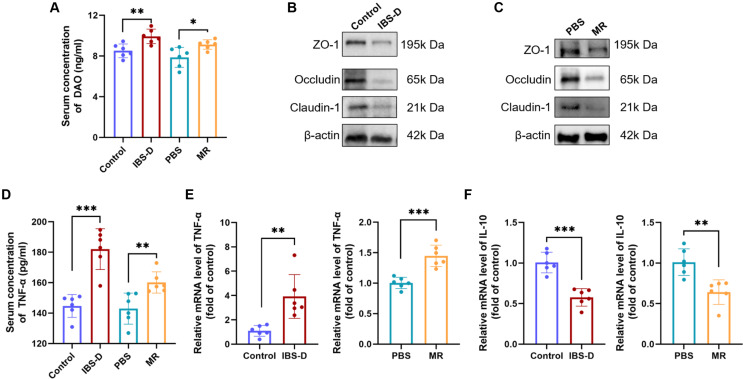
FMT-induced intestinal barrier impairment and low-grade inflammation in recipient rats. (**A**) Serum concentration of DAO (n = 6). (**B-C**) Expression of ZO-1, Occludin, and Claudin-1 proteins in colon. (**D**) Serum concentration of TNF-α (n = 6). (**E-F**) The mRNA levels of TNF-α and IL-10 in the colon (n = 6). Values are expressed as the mean ± SD; *p < 0.05, **p < 0.01, ***p < 0.001.

Altered intestinal barrier dysfunction and low-grade inflammation are recognized as key pathophysiological features of IBS-D [27]. To assess intestinal barrier integrity, we measured serum DAO levels and colonic tight junction (TJ) protein expression. Fig. 3A-3C shows that both the IBS-D and MR groups exhibited elevated serum DAO levels alongside reduced intestinal levels of the TJ proteins, including ZO-1, Occludin, and Claudin-1.

Low-grade inflammation is defined as unregulated and persistent synthesis and secretion of chemokines and cytokines. It is characterized by persistently elevated circulating levels of pro-inflammatory cytokines (particularly IL-6, TNF-α, and CRP) within 2 to 10 times the upper limit of the reference range observed in healthy individuals, without presenting clinical symptoms of acute local or systemic inflammation [28]. Serum concentration of the pro-inflammatory cytokine TNF-α was markedly elevated in the IBS-D and MR groups (Fig. 3D). In IBS-D rats, TNF-α mRNA expression in the colon was upregulated, while IL-10 mRNA expression was downregulated (Fig. 3E and 3F). Compared with the PBS group, TNF-α and IL-10 mRNA expression in the MR group exhibited similar directional changes (Fig. 3E and 3F). These results demonstrated that the MR group developed FMT-induced intestinal barrier dysfunction and low-grade inflammation, which were characterized by increased gut permeability, decreased TJ protein expression, and elevated levels of pro-inflammatory cytokines.

### Changes in Gut Microbiota Composition in IBS-D Donor and Recipient Rats

**Fig. 4:**
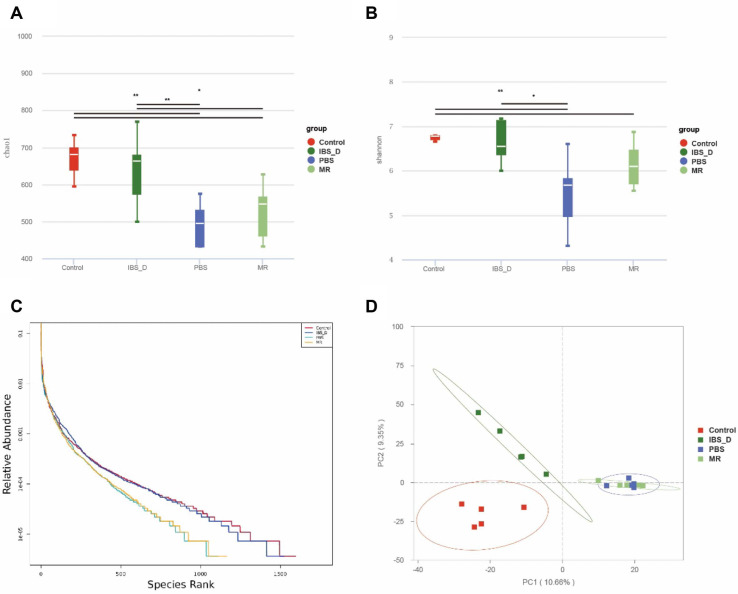
Diversity changes of gut microbiota (n = 5). (**A**) Shannon indexes. (**B**) Chao1 indexes. (**C**) Rank Abundance. (**D**) PCA score plots based on the ASVs level. Values are expressed as the mean ± SD; *p < 0.05, **p < 0.01.

To determine its association with the observed phenotypes, we characterized the gut microbiota composition via 16S rRNA gene sequencing. Both the PBS and MR groups showed a significant reduction in bacterial abundance, which may be attributed to the oral antibiotic treatment administered before FMT [29] (Fig. 4A-4C). Principal component analysis (PCA) revealed distinct microbial profiles between the IBS-D and control groups, while the MR group showed a compositional shift compared with the IBS-D group (Fig. 4D).

**Fig. 5:**
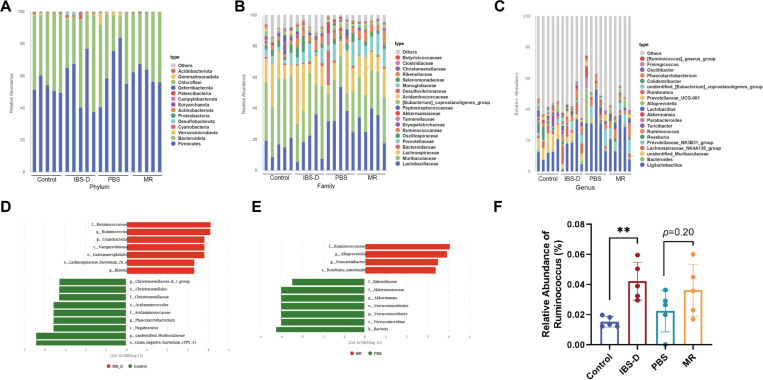
Gut microbiota composition in rats (n = 5). (**A-C**) Bacterial community composition at the phylum level(**A**), family level (**B**), genus level (**C**). (**D-E**) LDA value distribution histogram of IBS-D vs Control (**D**), MR vs PBS (**E**). (**F**) Relative abundance of *Ruminococcus*,**p < 0.01.

The gut microbiota in all groups was dominated by the *Firmicutes* and *Bacteroidetes*, as shown in Fig. 5A. The microbial composition at the family and genus levels could be found in Fig. 5B and Fig. 5C. Potential biomarkers across groups were identified by Linear Discriminant Analysis Effect Size (LEfSe) analysis (Fig. 5D and 5E). At an LDA threshold of 3, the IBS-D group exhibited a distinct microbial profile, characterized by the enrichment of the following top five taxa: *f_Ruminococcaceae*, *g_Ruminococcus*, *p_Cyanobacteria*, *c_Vampirivibrionia*, and *o_Gastranaerophilales*. Furthermore, comparison between the PBS and MR groups revealed four significantly more abundant taxa in the MR group: *f_Ruminococcaceae*, *g_Alloprevotella*, *g_Fusicatenibacter*, and *s_Roseburia_intestinalis*. Notably, *Ruminococcaceae* emerged as a key microbial in the pathogenesis of IBS-D, as evidenced by its high LDA scores and abundance in both the IBS-D and MR groups (Fig. 5D-5F).

### Metabolic Alterations across IBS-D Donor and Recipient Rats

**Fig. 6:**
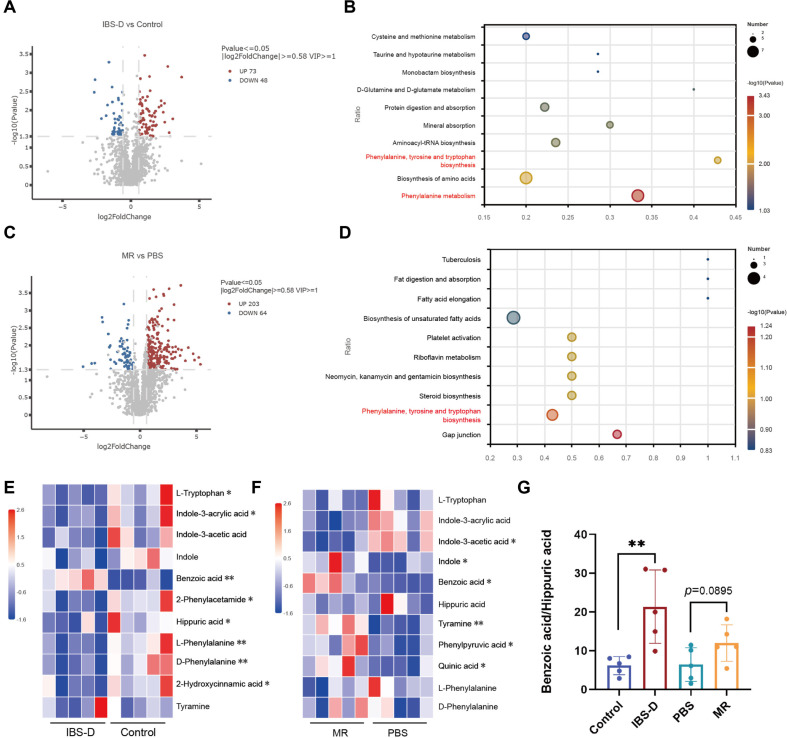
Comparative intestinal metabolite analysis in rats before and after FMT. (**A**) Volcano plot of the IBS-D group compared with the Control group. (**B**) Pathway enrichment analysis of altered metabolites between the IBS-D and Control groups. (**C**) Volcano plot of the MR group compared with the PBS group. (**D**) Pathway enrichment analysis of altered metabolites between the MR group and the PBS group. (**E-F**) Heatmaps overview of the differential metabolites in phenylalanine, tyrosine and tryptophan biosynthesis pathway that were alerted by the IBS-D group compared with the Control group (**E**) and the MR group compared with the PBS group (**F**). (**G**) The ratio of benzoic acid to hippuric acid. Values are expressed as the mean ±SD; *p < 0.05, **p < 0.01.

Metabolomic profiling was performed across groups to elucidate the functional impact of microbiota alterations. Partial Least Squares Discriminant Analysis (PLS-DA) was performed to assess the metabolic separation between groups (Fig. S1). Next, we screened the metabolites that were significantly altered based on the criteria of *p* ≤ 0.05, |log2FoldChange| ≥ 0.58, and VIP ≥ 1. Compared with the Control group, the IBS-D group exhibited 73 significantly upregulated and 48 downregulated metabolites (Fig. 6A). Comparative analysis between the MR and the PBS groups identified 203 significantly upregulated and 64 downregulated metabolites in the MR group (Fig. 6C). Subsequently, the KEGG database was used to annotate the enriched pathways of these altered metabolites. The pathway of Phenylalanine, tyrosine and tryptophan biosynthesis pathway was enriched not only between the IBS-D and Control groups, but also between the MR and PBS groups, indicating that the metabolites of these amino acids could play a role in the pathogenesis by action of FMT. Therefore, we next evaluated the metabolisms related to the Phenylalanine, tyrosine, and tryptophan biosynthesis pathway in samples of different groups. As shown in the heat maps (Fig. 6E), compared with the Control group, the IBS-D group exhibited 7 significantly downregulated and 1 significantly upregulated metabolite in this pathway. While the MR group exhibited an increase in 5 metabolites and a decrease in 1 metabolite compared with the PBS group (Fig. 6F). Meanwhile, we also found that levels of benzoic acid and the ratio of benzoic acid to hippuric acid were elevated both in the IBS-D and MR groups (Fig. 6G).

### The Potential Relationship between Metabolites and *Ruminococcus*

**Fig. 7:**
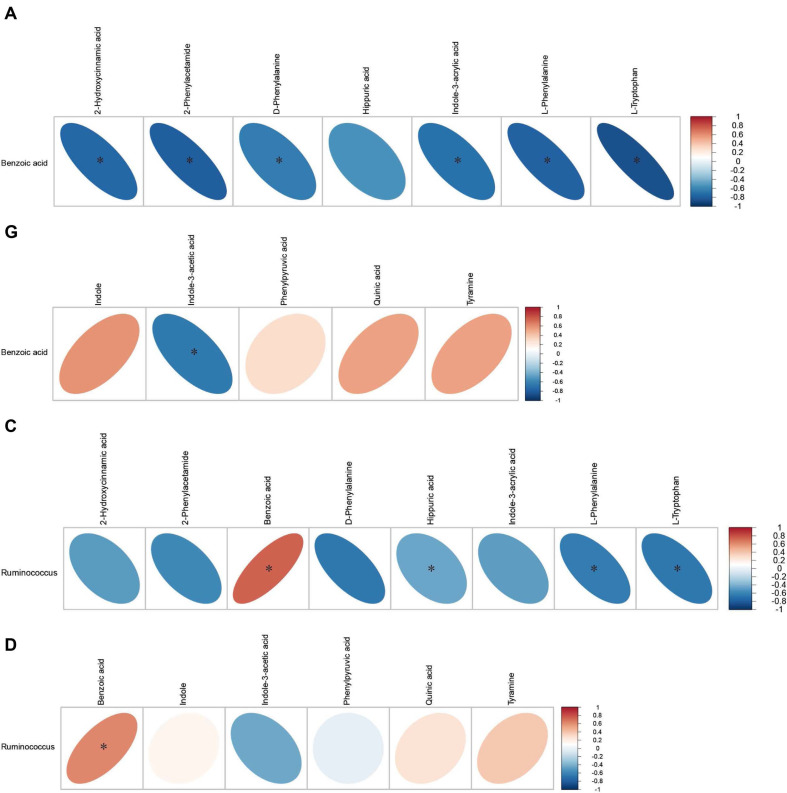
Correlation plot of the relative abundance of benzoic acid and other metabolites. (**A-B**) Pearson correlation analysis between benzoic acid and other significantly altered metabolites in phenylalanine, tyrosine and tryptophan biosynthesis pathway in the IBS-D vs. Control groups (**A**) and in the MR vs. PBS groups (**B**). (**C-D**) Pearson correlation analysis between *Ruminococcus* and significantly altered metabolites in phenylalanine, tyrosine and tryptophan biosynthesis pathway in the IBS-D vs. Control groups (**C**) and in the MR vs. PBS groups (**D**). The red spots indicate a positive correlation, while the blue ones show a negative correlation. The color intensity is proportional to the strength of the correlation, *p < 0.05.

Pearson correlation analysis was conducted to investigate the potential association between benzoic acid and other significantly altered metabolites in the phenylalanine, tyrosine and tryptophan biosynthesis pathway. In the IBS-D and Control groups, benzoic acid exhibited negative correlations with five amino acid metabolites, such as indole-3-acrylic acid and L-phenylalanine (Fig. 7A). In the MR and PBS groups, benzoic acid showed a negative correlation with indole-3-acetic acid (Fig. 7B). Between the IBS-D and Control groups (Fig. 7C), the *Ruminococcus*, a potential biomarker in IBS-D, was significant negatively correlated with L-phenyialanine (*p* = 0.047, R = -0.639) and L-tryptophan (*p* = 0.042, R = -0.650), while positively correlated with benzoic acid (*p* = 0.011, R = 0.757). Between the MR and PBS groups (Fig. 7D), the abundance of *Ruminococcus* positively correlated with benzoic acid (*p* = 0.047, R = 0.638).

## Discussion

Extensive research in recent years has explored the associations between gut microbiota alterations and their metabolites in IBS-D [13, 30]. While the gut microbiota and its metabolites are implicated in IBS-D progression, the precise mediators remain elusive. To this end, an IBS-D rat model was established using a previously reported method [20, 21]. This model successfully recapitulated key gastrointestinal and psychological features of IBS-D patients. Furthermore, our results show that FMT from IBS-D rats to healthy recipients significantly altered intestinal function, immune activation, and behavior, thereby suggesting that gut microbiota plays a causal role in IBS-D pathogenesis [31]. Next, we identified key candidates by analyzing the microbiota and microbiota-related metabolites. Integrated microbiome-metabolome analysis identified *Ruminococcus* and benzoic acid as consistently upregulated key candidates in IBS-D rats, consistent with prior studies[14, 32, 33]. Their levels were also elevated in post-FMT rats and showed a positive correlation. Our results indicated that *Ruminococcus*, a key pathogenic bacterium, along with benzoic acid, a critical metabolite, may jointly promote the progression of IBS-D.

Gut microbiota performs multiple essential functions through its roles in nutrient assimilation, immune regulation, and preservation of mucosal barrier function, thereby contributing crucially to host health [34]. In fact, multiple studies have evaluated alterations in the gut microbiota of IBS-D patients and confirmed that these changes are associated with disease phenotypes [13, 35]. Concurrently, FMT has gained traction both as a novel therapeutic intervention in clinical practice and as a robust methodological approach for studying microbiota-host interactions [36]. The successful phenotype transfer in our study strongly suggests that gut microbiota dysbiosis may play a causative role in the core symptoms of IBS-D, particularly the occurrence of intestinal barrier injury and low-grade inflammation in the intestine observed in patients [37]. This is consistent with previous studies, which showed that FMT from IBS-D patients to mice results in changes in gut transit, elevated permeability, and activation of intestinal immunity [18]. Importantly, these findings provide a mechanistic explanation for why microbiota-targeted therapies, such as probiotic supplementation [38, 39] and antibiotic treatment [40, 41], demonstrate clinical efficacy in IBS-D patients. Consequently, this study underscores the gut microbiota as a target for multimodal intervention strategies in IBS-D management.

We next focused on identifying microbial biomarkers responsible for the phenotypic changes mediated by FMT. Therefore, we also studied the changes in microbial composition through 16S rRNA sequencing technology. α-Diversity analysis showed no significant differences between the Control and IBS-D groups [15, 42], whereas β-diversity revealed distinct microbial community structures, indicating altered gut microbiota composition in IBS-D. Of note, both the PBS and MR groups exhibited significantly reduced bacterial abundance compared with the Control and the IBS-D groups, while β-diversity analysis showed significant compositional shifts in the MR group compared with the IBS-D group. These alterations are likely attributable to the prior establishment of pseudo-germ-free rat models leading to microbial depletion and incomplete microbial reconstitution post-FMT during the observation period [18, 43].

LEfSe analysis revealed that *Ruminococcus* significantly increased in relative abundance in both the IBS-D and MR groups, in agreement with previous reports [44]. Mounting evidence indicated that overgrowth of *Ruminococcus* was associated with intestinal disorders [45]. Research reported that *Ruminococcus* may be involved in the degradation of mucin, contributing to the damage of the intestinal barrier function in IBS-D [46]. Moreover, *Ruminococcus gnavus* (*R. gnavus*) produced inflammatory polysaccharides that induced dendritic cells to secrete pro-inflammatory cytokines such as TNF-α, potentially triggering inflammation in inflammatory bowel disease (IBD) [47]. The impact of *Ruminococcus* on IBS-D was further confirmed in independent studies. *R. gnavus* was significantly enriched in IBS patients, particularly IBS-D patients, correlating with elevated peripheral serotonin (5-HT) levels and symptom severity [14]. Therefore, our findings suggested that *Ruminococcus* may serve as a potential biomarker for IBS-D. These results provided valuable insights and laid the groundwork for developing microbiota-based diagnostic and therapeutic strategies.

Although the gut microbiota composition showed no significant differences between the PBS and the MR groups, their intestinal metabolite profiles underwent marked alterations. In-depth statistical analysis revealed that metabolites significantly altered in the MR and IBS-D groups showed increased in relative abundance in the phenylalanine, tyrosine, and tryptophan biosynthesis pathway, suggesting that these metabolites might be major contributors to the pathogenesis of IBS-D. Phenylalanine, tyrosine, and tryptophan are essential aromatic amino acids, which are the major nutrients and are closely linked to the intestinal barrier and immunity of the host. Gut microbiota metabolizes tryptophan into various indole derivatives, such as indole-3-acrylic acid and indole-3-acetic acid [48]. These compounds act as aryl hydrocarbon receptor (AhR) agonists, thereby contributing significantly to the maintenance of intestinal barrier integrity [49, 50]. Phenylalanine, 2-hydroxycinnamic acid, 2-phenylacetamide, phenylpyruvic acid, and benzoic acid belong to the phenylalanine biosynthesis pathway, which functions in synthesizing essential neurotransmitters and hormones [51]. While L- and D-phenylalanine differ in physiological activity, both can serve as substrates for bacterial decarboxylation, leading to tyramine production in the gut [52]. Tyramine induces intestinal contraction as well as inflammatory responses in intestinal epithelial cells and macrophages [53], and previous studies have reported elevated levels of tyramine in patients with IBS-D [30]. It is worth noting that the levels of benzoic acid were markedly elevated both in the IBS-D and the MR groups. Benzoic acid, a compound closely linked to the gut microbiota metabolism of dietary polyphenols [54-56], exerted beneficial effects on intestinal homeostasis, whereas excessive concentrations adversely impacted the gut lumen through luminal pH reduction and disruption of microbial colonization [57]. Besides, benzoic acid acts as an inhibitor of D-amino acid oxidase (DAAO), preventing degradation of D-amino acids—particularly D-serine [58, 59], which functions as a co-agonist of the N-methyl-D-aspartate receptor (NMDAR) and thereby promote central sensitization underlying chronic visceral hypersensitivity in IBS-D [60, 61]. Hippuric acid is considered a host-microbe cometabolite, as it is formed through the glycine conjugation of benzoic acid [62]. The elevated benzoic acid/hippuric acid ratio suggested impaired metabolisms of benzoic acid by the gut microbiota, leading to its subsequent accumulation [63, 64]. These impaired metabolisms of benzoic acid have also been reported in IBS patients [65]. These underlying mechanisms may contribute to the symptoms of IBS-D.

Correlation analysis could help us understand the complex relationships between microbiome and metabolome datasets [66, 67]. In this study, benzoic acid showed significant correlations with other altered metabolites in the phenylalanine, tyrosine, and tryptophan biosynthesis pathways, particularly with tryptophan-indole metabolites (indole-3-acrylic acid and indole-3-acetic acid). Shifts in the dominant gut microbiota may drive this pattern [13]. Besides, the abundance of *Ruminococcus* demonstrated a positive correlation with benzoic acid levels. However, although the direct mechanistic link between *Ruminococcus* and benzoic acid, as well as benzoic acid's pathophysiological impact on IBS-D, remained unresolved and merited further investigation, recent studies have indicated that *Ruminococcus* modulates the phenylalanine biosynthesis pathway to induce diarrheal symptoms in IBS patients [68]. *R. gnavus* catabolizes dietary phenylalanine and tryptophan to produce phenylethylamine and tryptamine, which activate TAAR1 to promote 5-HT biosynthesis in enterochromaffin cells, impair insulin signaling, and promote IBS-D progression [14] - a key focus of our future studies.

In summary, our study demonstrated that gut microbiota may contribute to IBS-D symptoms in recipient rats through multiple mechanisms, such as intestinal barrier disruption and immune dysregulation, as evidenced by FMT recipients from IBS-D rats similarly developing visceral hypersensitivity, diarrhea, mood disturbances, increased intestinal permeability, and low-grade inflammation. Concurrently, we identified *Ruminococcaceae* as a potential biomarker for both IBS-D donors and FMT recipients. Notably, benzoic acid levels showed a positive correlation with *Ruminococcus* abundance. This correlation suggested that benzoic acid may act as a key mediator of host alterations induced by this bacterium, although the underlying mechanism warrants further investigation. Our findings may provide foundations for targeting benzoic acid and *Ruminococcus* as both diagnostic biomarker and therapeutic target for IBS-D. However, several limitations of this study should be acknowledged. First, our experiments were conducted in rats, and although this model recapitulates key features of IBS-D, the findings may not fully translate to humans. Future studies with larger sample sizes or clinical investigations are needed to strengthen the conclusions. Second, the gut microbiota did not fully recover following antibiotic pretreatment, which may increase the risk of opportunistic pathogen overgrowth and could potentially influence intestinal function [69]. Third, the toxicological assessment and risk analysis of benzoic acid cannot be separated from its concentration, exposure context, and host factors. In this study, we only demonstrated a correlation between benzoic acid and *Ruminococcus* within the IBS-D microbiota. Therefore, future higher-resolution studies (e.g., metagenomics) are required to identify specific species and establish definitive causal relationships. Finally, a more in-depth elucidation of the underlying mechanisms and their clinical validation is warranted.

## Conclusion

FMT from IBS-D rats to healthy recipients recapitulated key disease features—including intestinal dysfunction, immune activation, and behavioral changes—confirming the pathogenic role of gut microbiota. Notably, both IBS-D and recipient rats exhibited a co-elevation of *Ruminococcus* abundance and benzoic acid levels, which were positively correlated. These findings suggested a potential association between *Ruminococcus* and benzoic acid, which may serve as microbial-metabolite correlates of IBS-D progression.

## Supplemental Materials

Supplementary data for this paper are available on-line only at http://jmb.or.kr.



## Figures and Tables

**Table 1:** Gene primer sequences for qRT-PCR.

Gene	Forward primer (5'-3')	Reverse primer (5'-3')
TNF-α	CGTCCCTCTCATACACTGGC	GCTTGGTGGTTTGCTACGAC
IL-10	TTGAACCACCCGGCATCTAC	CCAAGGAGTTGCTCCCGTTA
GAPDH	GGCACAGTCAAGGCTGAGAATG	ATGGTGGTGAAGACGCCAGTA

## References

[1] Greer K, Sultan S (2025). Irritable bowel syndrome. Ann. Intern. Med..

[2] Camilleri M (2021). Diagnosis and treatment of irritable bowel syndrome: a review. JAMA.

[3] Lacy BE, Pimentel M, Brenner DM, Chey WD, Keefer LA, Long MD (2021). ACG Clinical guideline: management of irritable bowel syndrome. Am. J. Gastroenterol..

[4] Oka P, Parr H, Barberio B, Black CJ, Savarino EV, Ford AC (2020). Global prevalence of irritable bowel syndrome according to Rome III or IV criteria: a systematic review and meta-analysis. Lancet Gastroenterol. Hepatol..

[5] Liu YL, Liu JS (2021). Irritable bowel syndrome in China: a review on the epidemiology, diagnosis, and management. Chin. Med. J. (Engl).

[6] Holtmann GJ, Ford AC, Talley NJ (2016). Pathophysiology of irritable bowl syndrome. Lancet Gastroenterol. Hepatol..

[7] Backhed F, Ley RE, Sonnenburg JL, Peterson DA, Gordon JI (2005). Host-bacterial mutualism in the human intestine. Science.

[8] Schaible P, Henschel J, Erny D (2025). How the gut microbiota impacts neurodegenerative diseases by modulating CNS immune cells. J. Neuroinflamm..

[9] Baars DP, Fondevila MF, Meijnikman AS, Nieuwdorp M (2024). The central role of the gut microbiota in the pathophysiology and management of type 2 diabetes. Cell Host Microbe..

[10] Li W, Gao W, Yan S, Yang L, Zhu Q, Chu H (2024). Gut microbiota as emerging players in the development of alcohol-related liver disease. Biomedicines.

[11] Schoultz I, Claesson M J, Dominguez-Bello M G, Fak Hallenius F, Konturek P, Korpela K (2025). Gut microbiota development across the lifespan: disease links and health-promoting interventions. J. Intern. Med..

[12] Cheng X, Ren C, Mei X, Jiang Y, Zhou Y (2024). Gut microbiota and irritable bowel syndrome: status and prospect. Front. Med. (Lausanne).

[13] Mars RAT, Yang Y, Ward T, Houtti M, Priya S, Lekatz HR (2020). Longitudinal multi-omics reveals subset-specific mechanisms underlying irritable bowel syndrome. Cell.

[14] Zhai L, Huang C, Ning Z, Zhang Y, Zhuang M, Yang W (2023). Ruminococcus gnavus plays a pathogenic role in diarrhea-predominant irritable bowel syndrome by increasing serotonin biosynthesis. Cell Host Microbe.

[15] Pittayanon R, Lau JT, Yuan Y, Leontiadis GI, Tse F, Surette M (2019). Gut microbiota in patients with irritable bowel syndrome-a systematic review. Gastroenterology.

[16] Mujagic Z, Kasapi M, Jonkers DM, Garcia-Perez I, Vork L, Weerts Z (2022). Integrated fecal microbiome-metabolome signatures reflect stress and serotonin metabolism in irritable bowel syndrome. Gut Microbes.

[17] El-Salhy M, Hatlebakk JG, Hausken T (2019). Diet in irritable bowel syndrome (IBS): interaction with gut microbiota and gut hormones. Nutrients.

[18] De Palma G, Lynch MD, Lu J, Dang VT, Deng Y, Jury J (2017). Transplantation of fecal microbiota from patients with irritable bowel syndrome alters gut function and behavior in recipient mice. Sci. Transl. Med..

[19] Shimbori C, De Palma G, Baerg L, Lu J, Verdu EF, Reed DE (2022). Gut bacteria interact directly with colonic mast cells in a humanized mouse model of IBS. Gut Microbes..

[20] Wei Y, Fan Y, Huang S, Lv J, Zhang Y, Hao Z (2024). Baizhu shaoyao decoction restores the intestinal barrier and brain-gut axis balance to alleviate diarrhea-predominant irritable bowel syndrome via FoxO1/FoxO3a. Phytomedicine.

[21] Sun J, Zhang M, Liu W, Liu Y, Zhang D, Fan X (2023). Evaluation of the effectiveness and mechanism of action of the Chang-Kang-Fang formula combined with bifid triple viable capsules on diarrhea-predominant irritable bowel syndrome. Front. Microbiol..

[22] Zhang M, Zheng Y, Li X, Wu H, Liu P, Zhang K (2022). Tong-Xie-Yao-Fang alleviates diarrhea-predominant irritable bowel syndrome in rats via the GCN2/PERK-eIF2alpha-ATF4 signaling pathway. Phytomedicine.

[23] Zhang S, Tian D, Xia Z, Yang F, Chen Y, Yao Z (2024). Chang-Kang-Fang alleviates diarrhea predominant irritable bowel syndrome (IBS-D) through inhibiting TLR4/NF-kappaB/NLRP3 pathway. J. Ethnopharmacol..

[24] Group FM T-s S (2020). Nanjing consensus on methodology of washed microbiota transplantation. Chin. Med. J. (Engl).

[25] Bokoliya SC, Dorsett Y, Panier H, Zhou Y (2021). Procedures for fecal microbiota transplantation in murine microbiome studies. Front. Cell. Infect. Microbiol..

[26] Yi X, Cai R, Shaoyong W, Wang G, Yan W, He Z (2023). Melatonin promotes gut anti-oxidative status in perinatal rat by remodeling the gut microbiome. Redox Biol..

[27] Ng QX, Soh A YS, Loke W, Lim D Y, Yeo WS (2018). The role of inflammation in irritable bowel syndrome (IBS). J. Inflamm. Res..

[28] Deng H, Chen Y, Xing J, Zhang N, Xu L (2024). Systematic low-grade chronic inflammation and intrinsic mechanisms in polycystic ovary syndrome. Front. Immunol..

[29] Ma P, Mo R, Liao H, Qiu C, Wu G, Yang C (2022). Gut microbiota depletion by antibiotics ameliorates somatic neuropathic pain induced by nerve injury, chemotherapy, and diabetes in mice. J. Neuroinflammation.

[30] Jacobs JP, Lagishetty V, Hauer MC, Labus JS, Dong TS, Toma R (2023). Multi-omics profiles of the intestinal microbiome in irritable bowel syndrome and its bowel habit subtypes. Microbiome.

[31] Weaver KR, Melkus GD, Henderson WA (2017). Irritable Bowel Syndrome. Am. J. Nurs..

[32] Baumgartner M, Lang M, Holley H, Crepaz D, Hausmann B, Pjevac P (2021). Mucosal Biofilms are an endoscopic feature of irritable bowel syndrome and ulcerative colitis. Gastroenterology.

[33] Zhai L, Huang C, Ning Z, Zhang Y, Zhuang M, Yang W (2023). Ruminococcus gnavus plays a pathogenic role in diarrhea-predominant irritable bowel syndrome by increasing serotonin biosynthesis. Cell Host Microbe.

[34] Kim S, Seo SU, Kweon MN (2024). Gut microbiota-derived metabolites tune host homeostasis fate. Semin. Immunopathol..

[35] Zhu S, Liu S, Li H, Zhang Z, Zhang Q, Chen L (2019). Identification of gut microbiota and metabolites signature in patients with irritable bowel syndrome. Front. Cell. Infect. Microbiol..

[36] El-Salhy M, Mazzawi T (2018). Fecal microbiota transplantation for managing irritable bowel syndrome. Expert Rev. Gastroenterol. Hepatol..

[37] Collins SM (2014). A role for the gut microbiota in IBS. Nat. Rev. Gastroenterol. Hepatol..

[38] Lenoir M, Wienke J, Fardao-Beyler F, Roese N (2025). An 8-Week course of bifidobacterium longum 35624((R)) is associated with a reduction in the symptoms of irritable bowel syndrome. Probiotics Antimicrob. Proteins.

[39] Xie P, Luo M, Deng X, Fan J, Xiong L (2023). Outcome-specific efficacy of different probiotic strains and mixtures in irritable bowel syndrome: a systematic review and network meta-analysis. Nutrients.

[40] Mokhtare M, Fathi M, Sadeghian A M, Sotoudeheian MJ, Namazi A (2024). A pilot study of the effectiveness of a short course of rifaximin 2200 mg/day on abdominal symptoms and its effects on quality of life in patients with moderate to severe diarrhea-predominant irritable bowel syndrome. Clin. Drug Investig..

[41] Mamieva Z, Poluektova E, Svistushkin V, Sobolev V, Shifrin O, Guarner F (2022). Antibiotics, gut microbiota, and irritable bowel syndrome: what are the relations?. World J. Gastroenterol..

[42] Sarnoff RP, Bhatt RR, Osadchiy V, Dong T, Labus JS, Kilpatrick LA (2023). A multi-omic brain gut microbiome signature differs between IBS subjects with different bowel habits. Neuropharmacology.

[43] Singh P, Alm EJ, Kelley JM, Cheng V, Smith M, Kassam Z (2022). Effect of antibiotic pretreatment on bacterial engraftment after fecal microbiota transplant (FMT) in IBS-D. Gut Microbes.

[44] Du L, Zhang Z, Zhai L, Xu S, Yang W, Huang C (2023). Altered gut microbiota-host bile acid metabolism in IBS-D patients with liver depression and spleen deficiency pattern. Chin. Med..

[45] La Reau AJ, Suen G (2018). The ruminococci: key symbionts of the gut ecosystem. J. Microbiol..

[46] Crost EH, Tailford LE, Monestier M, Swarbreck D, Henrissat B, Crossman L C (2016). The mucin-degradation strategy of Ruminococcus gnavus: the importance of intramolecular trans-sialidases. Gut Microbes.

[47] Henke MT, Kenny DJ, Cassilly CD, Vlamakis H, Xavier RJ, Clardy J (2019). *Ruminococcus gnavus*, a member of the human gut microbiome associated with Crohn's disease, produces an inflammatory polysaccharide. Proc. Natl. Acad. Sci. USA.

[48] Su X, Gao Y, Yang R (2022). Gut microbiota-derived tryptophan metabolites maintain gut and systemic homeostasis. Cells.

[49] Dong F, Perdew GH (2020). The aryl hydrocarbon receptor as a mediator of host-microbiota interplay. Gut Microbes.

[50] Li M, Ding Y, Wei J, Dong Y, Wang J, Dai X (2024). Gut microbiota metabolite indole-3-acetic acid maintains intestinal epithelial homeostasis through mucin sulfation. Gut Microbes.

[51] Fernstrom JD, Fernstrom MH (2007). Tyrosine, phenylalanine, and catecholamine synthesis and function in the brain. J. Nutr..

[52] Satoh Y, Fukui K, Koma D, Shen N, Lee TS (2023). Engineered Escherichia coli platforms for tyrosine-derivative production from phenylalanine using phenylalanine hydroxylase and tetrahydrobiopterin-regeneration system. Biotechnol Biofuels Bioprod..

[53] Bugda Gwilt K, Gonzalez D P, Olliffe N, Oller H, Hoffing R, Puzan M (2020). Actions of trace amines in the brain-gut-microbiome axis via trace amine-associated receptor-1 (TAAR1). Cell. Mol. Neurobiol..

[54] Del Olmo A, Calzada J, Nunez M (2017). Benzoic acid and its derivatives as naturally occurring compounds in foods and as additives: Uses, exposure, and controversy. Crit. Rev. Food Sci. Nutr..

[55] Nair B (2001). Final report on the safety assessment of Benzyl Alcohol, Benzoic Acid, and Sodium Benzoate. Int. J. Toxicol. 20 Suppl.

[56] Tanes C, Hu W, Friedman E, Hecht A, Daniel S, Clish C (2025). Distinguishing diet- and microbe-derived metabolites in the human gut. Microbiome.

[57] Choi H, Kim S W (2024). Dietary intervention of benzoic acid for intestinal health and growth of nursery pigs. Animals (Basel).

[58] Lin CH, Chen Y M, Lane HY (2020). Novel treatment for the most resistant schizophrenia: dual activation of NMDA receptor and antioxidant. Curr. Drug Targets..

[59] Lin CH, Lin CH, Chang YC, Huang YJ, Chen PW, Yang HT (2018). Sodium benzoate, a D-Amino acid oxidase inhibitor, added to clozapine for the treatment of schizophrenia: a randomized, double-blind, placebo-controlled trial. Biol. Psychiatry.

[60] Stanghellini V, De Ponti F, De Giorgio R, Barbara G, Tosetti C, Corinaldesi R (2003). New developments in the treatment of functional dyspepsia. Drugs.

[61] Yang Y, Wang J, Zhang C, Guo Y, Zhao M, Zhang M (2023). The efficacy and neural mechanism of acupuncture therapy in the treatment of visceral hypersensitivity in irritable bowel syndrome. Front. Neurosci..

[62] Pan Y, Yang Y, Peng Z, Wang W, Zhang J, Sun G (2025). Gut microbiota may modify the association between dietary polyphenol intake and serum concentrations of hippuric acid: results from a 1-year longitudinal study in China. Am J. Clin. Nutr..

[63] Ticinesi A, Guerra A, Nouvenne A, Meschi T, Maggi S (2023). Disentangling the complexity of nutrition, frailty and gut microbial pathways during aging: a focus on hippuric acid. Nutrients.

[64] Beyoglu D, Idle JR (2012). The glycine deportation system and its pharmacological consequences. Pharmacol. Ther..

[65] Kaczka A, Blonska A, Chojnacki C, Gasiorowska A, Blasiak J, Poplawski T (2025). Periodic changes in the gut microbiome in women with the mixed type of irritable bowel syndrome. Biomedicines.

[66] Tremaroli V, Backhed F (2012). Functional interactions between the gut microbiota and host metabolism. Nature.

[67] Fan Y, Pedersen O (2021). Gut microbiota in human metabolic health and disease. Nat. Rev. Microbiol..

[68] Zhai L, Xiao H, Lin C, Wong HLX, Lam YY, Gong M (2023). Gut microbiota-derived tryptamine and phenethylamine impair insulin sensitivity in metabolic syndrome and irritable bowel syndrome. Nat. Commun..

[69] Hong Y, Li H, Chen L, Su H, Zhang B, Luo Y (2024). Short-term exposure to antibiotics begets long-term disturbance in gut microbial metabolism and molecular ecological networks. Microbiome.

