# Supplementary material for: FMT from IBS-D Rats Impairs Intestinal Barrier Function and is Associated with Increased Intestinal Benzoic Acid and Ruminococcus Abundance
Source: J Microbiol Biotechnol. 2026 Jun 4;36:e2602004. doi: 10.4014/jmb.2602.02004 (PMC13275257; doi:10.4014/jmb.2602.02004)
Supplement: Supplementary file 1 [file jmb-36-e2602004-supple.pdf]

J. Microbiol. Biotechnol. 2026. 36: e2602004
https://doi.org/10.4014/jmb.2602.02004

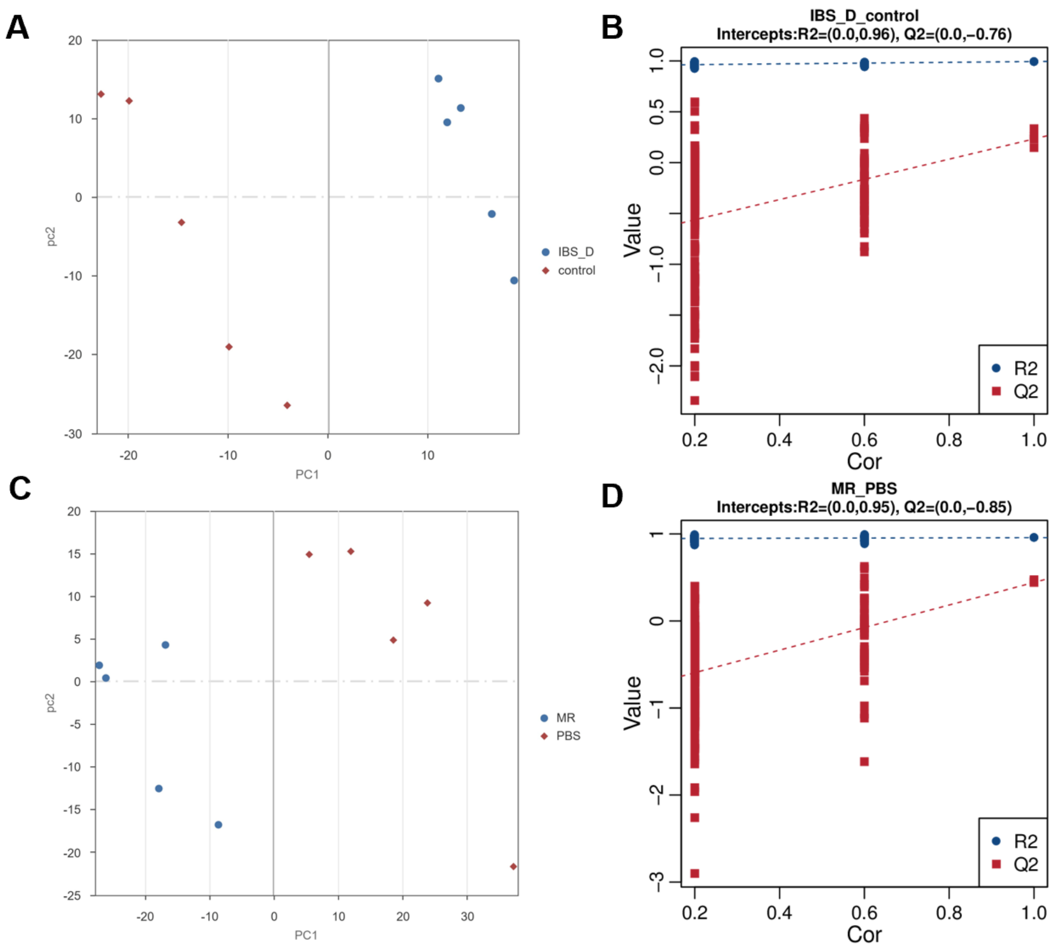

Fig. S1. FMT alters intestinal microbiota metabolic profiles in rats. (A-B) PLS-DA score plot and model validation for the Control vs. IBS-D groups. (C-D) PLS-DA score plot and model validation for the PBS vs. MR groups.
